# Determination of a Viral Load Threshold To Distinguish Symptomatic versus Asymptomatic Rotavirus Infection in a High-Disease-Burden African Population

**DOI:** 10.1128/JCM.00875-15

**Published:** 2015-05-14

**Authors:** A. Bennett, N. Bar-Zeev, K. C. Jere, J. E. Tate, U. D. Parashar, O. Nakagomi, R. S. Heyderman, N. French, M. Iturriza-Gomara, N. A. Cunliffe

**Affiliations:** a Malawi-Liverpool-Wellcome Trust Clinical Research Programme, College of Medicine, University of Malawi, Blantyre, Malawi; b Institute of Infection and Global Health, University of Liverpool, Liverpool, United Kingdom; c Centers for Disease Control and Prevention, Atlanta, Georgia, USA; d Department of Molecular Microbiology and Immunology, Nagasaki University, Nagasaki, Japan; e Liverpool School of Tropical Medicine, Liverpool, United Kingdom

## Abstract

We evaluated quantitative real-time PCR to establish the diagnosis of rotavirus gastroenteritis in a high-disease-burden population in Malawi using enzyme immunoassay as the gold standard diagnostic test. In 146 children with acute gastroenteritis and 65 asymptomatic children, we defined a cutoff point in the threshold cycle value (26.7) that predicts rotavirus-attributable gastroenteritis in this population. These data will inform the evaluation of direct and indirect rotavirus vaccine effects in Africa.

## TEXT

Rotavirus gastroenteritis is a major cause of infant morbidity and mortality with 90% of rotavirus-associated deaths occurring in low-income settings (1–3). Two live, oral vaccines (Rotarix [GlaxoSmithKline Biologicals, Belgium] and RotaTeq [Merck & Co. Inc., USA]) are entering childhood immunization programs worldwide. These have greatly reduced rotavirus-attributable hospitalizations in high-income and middle-income countries (4, 5), and a recently published study from Malawi was the first report of vaccine effectiveness from a low-income country (6).

Laboratory diagnosis of rotavirus gastroenteritis is typically established by detection of rotavirus antigen in stool using enzyme-immunoassay (EIA). This correlates well with clinical disease in developed settings but is less sensitive than real-time reverse transcription-quantitative PCR (qRT-PCR) (7, 8). However, as qRT-PCR can detect rotavirus shedding in the stool in up to 30% of asymptomatic young children in developed settings, the clinical implications of a positive result is uncertain (9, 10). A positive relationship between fecal viral load and EIA-positive rotavirus disease has been demonstrated in children from the United Kingdom and the United States (7, 11) and has been used to define a quantitative cycle threshold (C_*T*_) cutoff point in qRT-PCR corresponding to clinical disease (7, 11).

Much of our understanding of the performance of rotavirus diagnostic tests comes from well-resourced settings (11–13). However, rotavirus disease patterns in the poorest settings differ from those in the developed world, with higher force of infection, earlier disease onset, and delayed acquisition of immunity, which may impact diagnosis (14, 15). This study in Blantyre, southern Malawi, aimed to evaluate the performance of qRT-PCR and EIA in order to define a cutoff point in the qRT-PCR *C_T_* value to define clinical disease in a high-burden, resource-poor African country and was undertaken as part of an assessment of monovalent rotavirus vaccine effectiveness following its introduction into Malawi's national immunization program in October 2012 (6). Children <5 years of age were recruited on presentation to Queen Elizabeth Central Hospital (QECH) with acute diarrhea (the passage of three or more loose stools within a 24-h time period within 14 days of presentation). Age- and neighborhood-matched community controls were enrolled in Blantyre District (6). A single stool sample was collected from each child and tested for rotavirus antigen by EIA (Premier Rotaclone; Meridian Biosciences, Cincinnati, OH) without knowledge of clinical status.

Following nucleic acid extraction from 10% to 20% stool suspensions, cDNA was synthesized using random primers. VP6 qRT-PCR was performed using VP6 gene-specific primers and probes as previously described, using 2× TaqMan Universal Master Mix II (Invitrogen, Paisley, United Kingdom), and a Rotor-Gene Q 5-plex system (Qiagen, Manchester, United Kingdom) (13, 16). A rotavirus qRT-PCR result was deemed positive if the *C_T_* value was <40 and negative when the C_*T*_ value was ≥40.

For amounts of cDNA, >30 copies intra-assay reproducibility was high (Table 1). Samples positive by qRT-PCR that had a *C_T_* value below 11 (*n* = 2) could not be quantified.

**TABLE 1:** VP6 qPCR inter-assay reproducibility in eight separate runs

Standard curve input copy no.	C*_T_* value			Calculated copy no.	
	Mean	Minimum	Maximum	Mean	%CV^*a*^
3,000,000	15.08	14.86	16.35	2,929,261	0.13
300,000	18.91	18.14	19.25	335,457	0.12
30,000	23.34	22.29	24.99	27,236	0.14
3,000	26.85	25.90	27.73	3,320	0.20
300	31.47	30.51	32.88	306	0.20
30	34.94	33.45	35.98	30	0.15
3	38.23	37.27	>40 (neg)	4^*b*^	0.25^*b*^

a CV, coefficient of variation.

b Calculated only for assays in which the result was positive for this sample (<40).

Frequency of detection of rotavirus using EIA and qRT-PCR was described and tested for discordance using McNemar's test. Median *C_T_* values were compared between EIA-positive (diarrhea), EIA-negative (diarrhea), and asymptomatic samples using Wilcoxon rank sum tests. Receiver operating characteristics (ROC) analysis was used to evaluate the ability of qRT-PCR *C_T_* values to discriminate between the presence and absence of symptomatic gastroenteritis, using EIA as a gold standard (17, 18) and defining the absence of disease as EIA negative without diarrhea. A cutoff point that optimized sensitivity and specificity was identified using Youden's index [(sensitivity + specificity) − 1] (19, 20). Analysis was conducted using Stata 12.1 (StataCorp, USA) and GraphPad Prism 6 (GraphPad Software, Inc., USA). Ethical approval was provided by the National Health Sciences Research Committee, Lilongwe, Malawi (867) and by the Research Ethics Committee of the University of Liverpool, Liverpool, United Kingdom (000490).

A total of 238 fecal samples were collected. Of these, clinical data were available for 225. Fourteen community samples were excluded for intercurrent diarrhea, resulting in 211 samples available for analysis: 77 EIA-positive diarrheal cases, 69-EIA negative diarrheal cases, and 65 asymptomatic children. Of the 211 samples with corresponding clinical data, 77 of the 77 (100%) children with EIA-positive diarrhea were also qRT-PCR positive and 34 (49.3%) of 69 children with EIA-negative diarrhea were qRT-PCR positive. Of the 65 asymptomatic community controls, 1 (1.5%) was EIA positive and 20/65 (30.8%) had virus detected using qRT-PCR.

**FIG 1:**
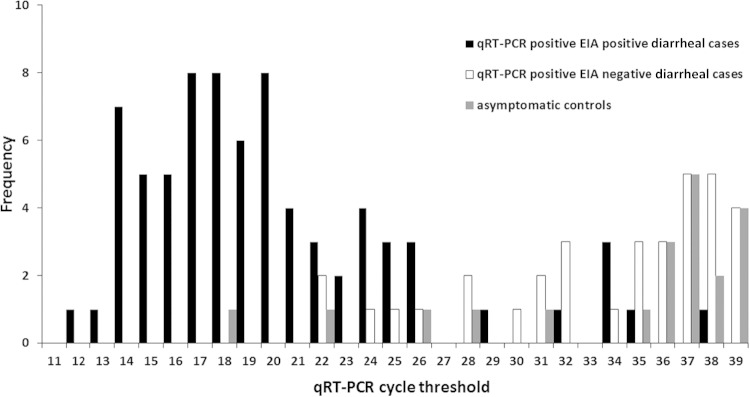
Distribution of qRT-PCR *C_T_* values between diarrheal cases and asymptomatic controls.

**FIG 2:**
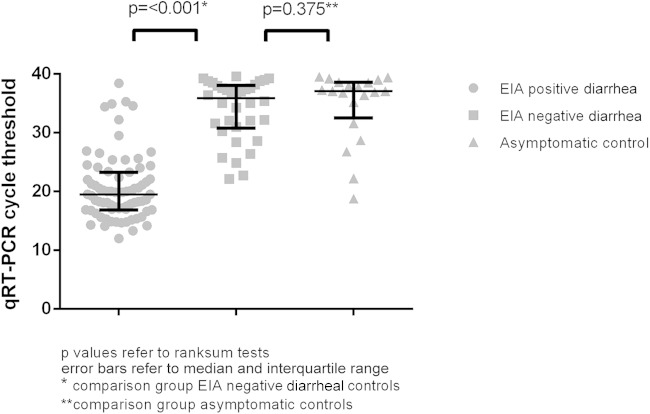
qRT-PCR *C_T_* values according to EIA result and clinical category.

There was a significant difference in median qRT-PCR *C_T_* values between EIA-negative diarrheal cases (median, 35.9; interquartile range [IQR], 31.0 to 38.0) and those of EIA-positive diarrheal cases (median, 19.5; IQR, 16.9 to 23.3; *P* = <0.001), but there was no significant difference in *C_T_* values between EIA-negative diarrheal cases and those of asymptomatic community controls (median, 37.1; IQR, 33.4 to 38.6; *P* = 0.375) (Fig. 1 and 2). Quantitative real-time PCR *C_T_* values discriminated well between the presence and absence of rotavirus gastroenteritis as defined using EIA, with an area under the curve of 0.96 (95% confidence interval [CI], 0.93 to 1.00). The point that maximized the Youden index was at a *C_T_* value of 26.7, at which point sensitivity and specificity were 89% and 95%, respectively (corresponding with an average cDNA copy number per reaction of 3,000 [95% CI, 2,657 to 3,514]; Table 1).

We have demonstrated differences in *C_T_* values between rotavirus EIA-positive and EIA-negative diarrheal children in a high-burden, low-resource setting and defined an optimal C_*T*_ cutoff point, which may be utilized to diagnose clinical disease as molecular diagnostics are increasingly rolled out in this region. We found that, consistent with data from the United Kingdom and the United States, a positive rotavirus EIA result was strongly associated with the presence of diarrhea, with only one asymptomatic, EIA-positive child identified (7, 11). However, qRT-PCR was more sensitive at detecting rotavirus in stool than EIA (12), with a high frequency (31%) of low-level viral shedding detected by qRT-PCR in samples from the asymptomatic group. This confirms that detection of rotavirus using qRT-PCR without consideration of viral load is insufficient to attribute disease causation (11). The prevalence of viral shedding in the asymptomatic group is high (31%) but is consistent with that found in young children in the United Kingdom (9).

There were significant differences in the rotavirus *C_T_* values between diarrheal children who were EIA positive and those who were EIA negative, with substantially higher *C_T_* values (lower viral load) in children who were EIA negative, and no significant difference in qRT-PCR *C_T_* values between EIA-negative diarrheal children and those of asymptomatic children. ROC analysis confirmed the discriminatory value of qRT-PCR *C_T_* values. The cutoff point in our study is similar to that defined in a United Kingdom population using the same assay (11), suggesting that any difference in frequency of infection or in disease severity between the two settings does not impact distribution of viral loads and that EIA and qRT-PCR perform similarly in the detection of rotavirus gastroenteritis in each environment.

Children were recruited from a referral hospital, and a large number had severe disease. However, the cutoff we identified correlates well with that identified using a cohort of children recruited from primary care in the United Kingdom, in whom less severe disease should be better represented (11). Moreover, since reaction sensitivities may vary between PCR assays conducted in different laboratories, we have provided cDNA copy numbers corresponding to the qRT-PCR *C_T_* values to facilitate comparison.

Assessment of rotavirus vaccine effectiveness requires accurate laboratory diagnostics. In high-burden, low-income settings where direct vaccine effectiveness is lower (6), demonstrating indirect (herd) benefits becomes more important. Should rotavirus vaccine reduce asymptomatic shedding, its impact on community rotavirus transmission may be substantial. Future evaluation of vaccine indirect effects will require measurement of impact on asymptomatic shedding and community transmission using the cutoffs we have defined in this paper. Our findings, therefore, inform the evaluation of direct and indirect rotavirus vaccine effects in Africa.
